# Does Single Dose Epinephrine Improve Outcomes for Patients with Out-of-Hospital Cardiac Arrest by Sex or Race?

**DOI:** 10.5811/westjem.41482

**Published:** 2025-09-25

**Authors:** Breanna L. Blaschke, Nicklaus P. Ashburn, Anna C. Snavely, Kristina Dev, Tyler S. George, Bryan P. Beaver, Michael A. Chado, Harris A. Cannon, James E. Winslow, R. Darrell Nelson, Jason P. Stopyra, Simon A. Mahler

**Affiliations:** * University of North Carolina, Department of Emergency Medicine, Chapel Hill, North Carolina; † Wake Forest University School of Medicine, Department of Emergency Medicine, Winston-Salem, North Carolina; ‡ Wake Forest University School of Medicine, Department of Biostatistics and Data Science, Winston-Salem, North Carolina; § Wake Forest University School of Medicine, Department of Internal Medicine, Winston-Salem, North Carolina; || University of Kansas School of Medicine, Department of Emergency Medicine, Kansas City, Kansas; # The Ohio State University, Department of Emergency Medicine, Columbus, Ohio; ¶ Wake Forest University School of Medicine, Department of Epidemiology and Prevention, Winston-Salem, North Carolina; ** Wake Forest University School of Medicine, Department of Implementation Science, Winston-Salem, North Carolina

## Abstract

**Introduction:**

Recent evidence suggests that survival to hospital discharge in patients with out-of-hospital cardiac arrest (OHCA) is similar among patients receiving a single dose epinephrine protocol compared to a multi-dose epinephrine protocol. However, it is unknown whether survival to hospital rates differ for single dose vs. multi-dose epinephrine within sex and race subgroups. Our objective in this study was to determine whether survival to hospital discharge rates varied for single dose vs. multi-dose epinephine protocols among men, women, White, and non-White patients.

**Methods:**

We conducted a pre-post Single Dose Epinephrine Implementation Study from November 1,2016 – October 29, 2019 at five North Carolina emergency medical services (EMS) systems, involving patients ≥ 18 years old with non-traumatic OHCA. Data on race, sex, and the primary outcome of survival to hospital discharge were determined from the Cardiac Arrest Registry to Enhance Survival and from EMS records. We performed intention-to-treat analysis. We compared survival to hospital discharge rates between single dose vs multi-dose epinephrine protocols within sex and race subgroups using generalized estimating equations with a logit link to account for clustering among EMS agencies and to adjust for age, witnessed arrest, automated external defibrillator availability, EMS response interval, the presence of a shockable rhythm, receiving bystander cardiopulmonary resuscitation, and sex or race. In the model, we evaluated interactions between epinephrine protocol and race and sex.

**Results:**

Of the 1,690 patients included, (899 multi-dose, 791 single dose), 38.7% (657/1,690) were female and 74.7% (1,262/1,690) were White. Survival to hospital discharge occurred in 13.6% (122/899) of patients in the multi-dose group and 15.4% (122/791) in the single dose epinephrine group (OR 1.19, 95%CI 0.89–1.59). Single dose epinephrine was associated with increased survival to hospital discharge rates in White patients (adjusted odds ratio [aOR] 1.17, 95% confidence interval [CI] 1.05–1.30). However, the rates were similar for single dose vs. multi-dose epinephrine among men (aOR 1.03, 95% CI 0.93–1.14), women (aOR 1.23, 95% CI 0.97–1.56), and non-White patients (aOR 1.08, 95% CI 0.78–1.51). Interactions between epinephrine protocol and subgroups were not significant.

**Conclusion:**

Rates of survival to hospital discharge were similar in the single dose and multi-dose epinephrine strategies regardless of sex. Single dose epinephrine was associated with increased survival to hospital discharge among White patients but not in non-White patients, which may be due to unmeasured confounding or inadequate power.

## INTRODUCTION

In the United States approximately 350,000 patients experience out-of-hospital cardiac arrest (OHCA) annually, and only about 10% survive.^1^–^3^ Per the current Advanced Cardiovascular Life Support (ACLS) guidelines set by the American Heart Association, epinephrine should be administered every three to five minutes for OHCA as part of a multi-dose epinephrine protocol.^4^ However, while epinephrine is known to increase return of spontaneous circulation (ROSC) rates, it may not improve rates of survival to hospital discharge.^5^–^7^ Increased myocardial oxygen demand and risk of cardiac arrhythmias associated with epinephrine may contribute to adverse outcomes and decrease survival to hospital discharge.^8^–^10^

Our team recently implemented a single dose epinephrine protocol for OHCA in five emergency medical services (EMS) agencies. When compared with the traditional multi-dose protocol, we found that the single dose protocol was associated with decreased rates of ROSC but similar rates of survival to hospital discharge.^11^ Prior literature has established that cardiovascular care disparities exist between men and women and White and non-White patients.^2^,^12^–^18^ However, no study has compared the performance of single dose vs. multi-dose epinephrine approach within sex and race subgroups.

To address this gap in the out-of-hospital resuscitation literature, we conducted a pre-planned secondary analysis of the Single dose Epinephrine Implementation Study, which compared adult OHCA patients receiving resuscitation guided by single dose vs. multi-dose epinephrine. Our objectives were to determine whether survival to hospital discharge, ROSC, and favorable neurologic outcome rates differed for single dose vs. multi-dose epinephrine protocols among men, women, White, and non-White patients. We also explored survival to hospital discharge, ROSC, and favorable neurologic outcomes among patients presumed to have arrested from a primary cardiac etiology.

## METHODS

### Study Design and Oversight

We performed a secondary analysis of the Single Dose Epinephrine Implementation Study for OHCA. Data were collected from five EMS systems in North Carolina between November 1, 2016 – October 29, 2019. The Wake Forest University Health Sciences Institutional Review Board approved the study protocol and granted a waiver of informed consent. We used the Strengthening the Reporting of Observational Studies in Epidemiology guidelines for reporting observational studies during the research process.^19^ The methods of the Single dose Epinephrine Implementation Study have been previously described.^11^,^20^

### Study Setting and Population

Five counties were included in the study, each operating an Advanced Life Support (ALS) EMS system with medical direction from emergency physicians with subspecialty board certification in EMS. These EMS systems serve populations within urban, suburban, and rural communities, totaling almost 850,000 individuals (Supplemental Table 1). Patients ≥ 18 years of age who underwent attempted resuscitation for non-traumatic cardiac arrest were included in the study. We excluded patients who were pregnant and prisoners. Pre-implementation, the traditional multi-dose epinephrine recommendations were followed. Under these guidelines, EMS responders administered 1 milligram (mg) of 1:10,000 intravenous (IV) or intraosseous (IO) epinephrine every 3–5 minutes. Post-implementation, responders followed the single dose epinephrine protocol, where patients received a single 1:10,000 dose of 1 mg IV or IO epinephrine. The protocol did not allow for additional doses of epinephrine. During the post-implementation period, the North Carolina Office of EMS also began allowing the use of ketamine for cardiopulmonary resuscitation (CPR)-induced consciousness. Two patients received ketamine for this indication over the study period. No other changes were made to the study protocol. The complete single dose and multi-dose epinephrine guidelines are found in Supplemental Appendix 1.

**Population Health Research Capsule**

What do we already know about this issue?

*It is unknown whether survival to hospital-discharge rates differ for a single dose vs. multi-dose epinephrine protocol within sex and race subgroups*.

What was the research question?

*Do rates of survival to hospital discharge vary for single dose vs. multi-dose epinephrine protocols among men, women, White, and non-White patients?*

What was the major finding of the study?

*Single dose epinephrine was associated with increased survival to hospital discharge in White patients (aOR 1.17, 95% CI 1.05–1.30)*.

How does this improve population health?

*Rates of survival to hospital discharge were similar in the single dose and multi-dose groups regardless of sex. Single dose was associated with increased survival among White patients only*.

### Data Collection and Variables

We collected data for one year before and one year after the date of single dose epinephrine implementation, which differed for each county EMS system. Exact dates of implementation for each EMS system are included in Supplemental Table 1. All data were collected from November 1, 2016 – October 29, 2019.

We used the Cardiac Arrest Registry to Enhance Survival (CARES) to collect demographics, initial heart rhythm, interventions provided, and etiology of the arrest.^21^ The EMS systems submit data to CARES using standardized international Utstein definitions to help ensure uniformity in reporting.^21^ Sex and race were determined by the prehospital responder based on patient or family report, driver’s license, or by healthcare clinician impression. Race data in CARES was recently validated in a large cohort of Medicare data.^22^ The CARES registry defined shockable rhythms as ventricular fibrillation, ventricular tachycardia, and unknown shockable rhythm. Non-shockable rhythms were defined as asystole, idioventricular/pulseless electrical activity, and unknown unshockable rhythm. Per CARES, the etiology of the arrest was presumed to be cardiac, unless it was likely from a known respiratory cause, asphyxia, drowning, or electrocution. The CARES registry also provided patient outcomes, including survival to hospital discharge, ROSC, and neurologic status. When abstracting data from CARES and the prehospital electronic health record, we used best practices to enhance scientific rigor. We used trained data abstractors, case selection criteria, variable definitions, performance monitoring, an electronic abstraction form, and medical record identification.^23^

### Outcomes

The primary outcome was survival to hospital discharge, defined by CARES as leaving the hospital alive regardless of neurologic status. The secondary outcome was ROSC, defined as the patient having a pulse for ≥ 20 minutes without additional chest compressions.^21^ An exploratory outcome was neurological outcome at time of discharge from the hospital. The CARES registry describes neurologic outcomes using Cerebral Performance Categories (CPC) 1–4, with categories 1 and 2 considered favorable due to patients being able to function independently and live a normal, or relatively normal, life. The CPC 3 and 4 are considered poor neurological outcomes, with patients having severe cerebral disability or being in a vegetative state, requiring daily support due to impaired brain function. The CARES registry does not use the CPC 5 (brain death) category.

### Statistical Analysis

We described categorical variables such as sex, race, EMS system, rhythm types, performance of bystander CPR, as well as survival to hospital discharge and ROSC rates, with counts and percentages. Continuous variables, such as age and EMS response time, were described with medians and interquartile ranges (IQR). The unit of analysis was the OHCA encounter, and the analysis was by intention to treat. It was not possible to conduct a per-protocol analysis because the number of doses of epinephrine administered was not available. We compared survival to hospital-discharge rates, ROSC rates, and neurological outcomes between the single dose and multi-dose epinephrine cohorts using generalized estimating equations (GEE) with a logit link to account for clustering within EMS agencies.

Models were fit within each subgroup, defined by sex (male or female) and race (White or non-White). Non-White patients were those who identified as American Indian/Alaska Native, Asian, Black, Hispanic/Latino, or Native Hawaiian/Pacific Islander. Due to the study’s modest sample size, we analyzed race as a two-level variable: White and non-White. This dichotomous approach has been used in prior cardiovascular care studies.^24^–^28^ We also evaluated the interaction of single dose epinephrine implementation (multi-dose vs. single dose epinephrine) with sex and race. Multivariable models were adjusted for age, witnessed arrest, automated external defibrillator (AED) availability, EMS response interval, presence of a shockable rhythm, receiving bystander CPR, and sex or race. Unadjusted and adjusted odds ratios (aOR) with corresponding 95% confidence intervals (CI) were calculated from the GEE models. Using the same analysis methods described above, we also conducted pre-specified analyses among only those patients who experienced OHCA from a presumed primary cardiac etiology.

## RESULTS

**Figure 1:**
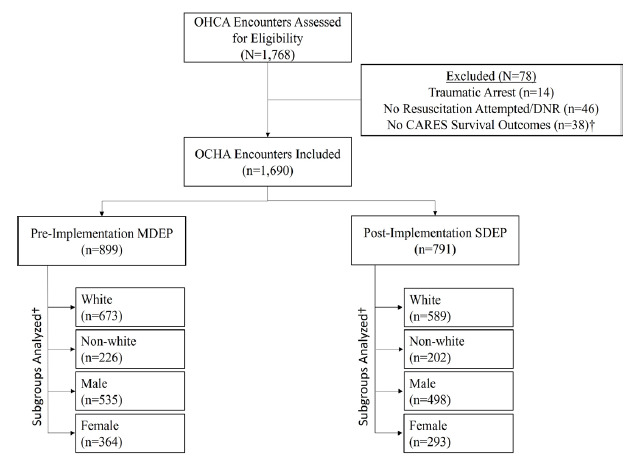
The study flow diagram. ^†^While 38 total encounters were without CARES outcomes, only 18 additional encounters were excluded once encounters for traumatic arrest and those with no resuscitation attempted/DNR were excluded. *OHCA*, out-of-hospital cardiac arrest; *DNR*, do not resuscitate; *CARES*, Cardiac Arrest Registry to Enhance Survival; *MDEP*, multidose epinephrine protocol; *SDEP*, single-dose epinephrine protocol.

During the study period there were 1,690 OHCA encounters (899 pre-implementation with multi-dose epinephrine, 791 post-implementation with single dose epinephrine). The overall cohort was 74.7% White (1,262/1,690) and 38.9% female (657/1,690); the median age was 65 years (IQR 53–76). Survival to hospital discharge occurred in 13.6% (122/899) of patients in the multi-dose epinephrine group and 15.4% (122/791) in the single dose epinephrine group (OR 1.19, 95%CI 0.89–1.59),^11^ while ROSC occurred in 42.3% (380/899) of patients in the multi-dose epinephrine group and 32.5% (257/791) in the single dose epinephrine group. Figure 1 illustrates the study flow diagram. Patient characteristics are summarized in Table 1.

**Figure 2:**
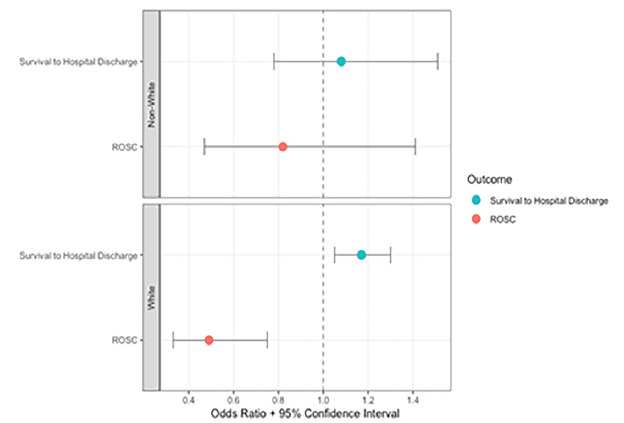
Single dose vs. multi-dose epinephrine adjusted odds ratios for study outcomes among all White vs non-White patients. Models were adjusted for age, witnessed arrest, location of the arrest, automatic external defibrillator availability, emergency medical services response interval, the presence of a shockable rhythm, receiving bystander cardiopulmonary resuscitation, and sex. *ROSC*, return of spontaneous circulation.

Single dose epinephrine implementation was associated with a 1.9% absolute increase in survival to hospital discharge among White patients (15.1% vs. 13.2%; OR 1.18, 95% CI 1.06–1.31) and a 1.7% increase among non-White patients (16.3% vs. 14.6%; OR: 1.17, 95% CI 0.68–2.02). After adjusting for potential confounders, single dose epinephrine implementation remained associated with higher survival to hospital-discharge rates among White patients (aOR 1.17, 95% CI 1.05–1.30) but not among non-White patients (aOR 1.08, 95% CI 0.78–1.51). Single dose epinephrine was associated with decreased ROSC rates among White patients (aOR 0.49, 95% CI 0.33–0.75), but relatively unchanged ROSC rates among non-White patients (aOR 0.82, 95% CI 0.47–1.41). The interaction between single dose epinephrine implementation and race was not significant for survival to hospital discharge (*P* = .84) or ROSC (*P* = .13). Table 2 and Figure 2 show the study outcomes by race. The exploratory outcome of favorable neurologic status is presented in Table 2.

**Figure 3:**
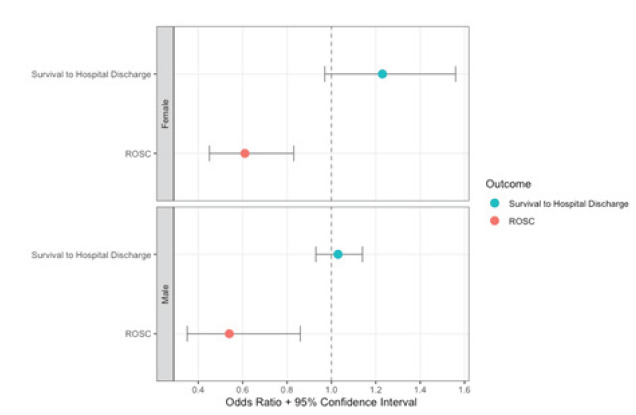
Single dose vs. multi-dose epinephrine adjusted odds ratios for study outcomes among all male vs female patients. Models were adjusted for age, witnessed arrest, location of the arrest, automatic external defibrillator availability, emergency medical services response interval, the presence of a shockable rhythm, receiving bystander cardiopulmonary resuscitation, and race. *ROSC*, return of spontaneous circulation; *CPR*, cardiopulmonary resuscitation.

Survival to hospital discharge rates did not differ between the single dose and multi-dose epinephrine periods among women (16.7% vs. 13.7%; OR 1.28, 95% CI 0.91–1.80) or men (14.7% vs. 13.5%; OR: 1.11, 95% CI 0.97–1.28). When adjusting for potential confounders, similar survival to hospital discharge rates persisted among women (aOR 1.23, 95% CI 0.97–1.56) and men (aOR 1.03, 95% CI 0.93–1.14). However, single dose epinephrine was associated with decreased ROSC in women (aOR 0.61, 95% CI 0.45–0.83) and men (aOR 0.54, 95% CI 0.35–0.83). The interaction between single dose epinephrine implementation and sex was not significant for survival to hospital-discharge rates (*P* = .47) or ROSC (*P* = .31). Table 3 and Figure 3 show the study outcomes by sex. The exploratory outcome of favorable neurologic status is presented in Table 3.

Among patients thought to have arrested from a primary cardiac etiology, we found that survival to hospital-discharge rates improved among non-White patients (aOR 1.22, 95%CI 1.01–1.48); however, single dose epinephrine was no longer associated with improved survival to hospital-discharge rates among White patients (aOR 1.28, 95%CI 0.84–1.95). Similar to the overall group, survival to hospital-discharge rates were similar between single dose and multi-dose epinephrine among men (aOR 1.17, 95% CI 0.73–1.87) and women (aOR 1.35, 95%CI 0.97–1.91) thought to have arrested from a primary cardiac etiology. Supplemental Tables 2–3 and Supplemental Figures 1–2 present study outcomes in these pre-specified subgroups.

## DISCUSSION

The key finding of this subgroup analysis is that implementation of a single dose epinephrine protocol was associated with similar survival to hospital-discharge rates among men, women, and non-White patients. While we did detect an improvement in survival to hospital-discharge rates among White patients, we suspect that this was likely driven by unmeasured confounders and social determinants of health. We do not believe that there is a meaningful, biologically plausible reason for the single dose epinephrine protocol to improve outcomes among White patients but not among other key subgroups.^17^,^18^

Prior research has found that Black patients and other racial minority patients are more likely to suffer poorer outcomes from OHCA compared to White patients, including worse rates of survival - hospital discharge and neurologic recovery.^15^,^16^ This has been previously associated with several factors, including lower rates of bystander CPR and defibrillator use in non-White compared to White patients.^15^,^16^ No study to date has explored the effect of single dose vs. multi-dose epinephrine in race subgroups. In this analysis we found that White patients had improved survival to hospital-discharge rates with the single dose epinephrine protocol while non-White patients had no significant difference in survival to hospital-discharge rates. Given that nearly 75% of our sample was comprised of White patients and that the point estimates for survival to hospital discharge with single-does epinephrine implementation were similar for White and non-White patients, we believe that our study was likely underpowered to detect a significant difference in survival to hospital discharge among non-White patients.

Furthermore, among the patients thought to have arrested from a primary cardiac etiology, we observed the opposite: non-White patients had improved survival to hospital discharge while White patients had similar rates of survival to hospital discharge. The difference in findings between all patients compared to just those with presumed arrest from a cardiac etiology suggests the possibility that there might not be an association at all between single dose epinephrine implementation and survival to hospital discharge. Our findings should be cautiously interpreted: We do not think there is a causative physiologic reason driving these differences in outcomes. Rather, it is more likely that race is a marker of risk, which may be driven by socioeconomic, environmental, or access-to-care factors.

Prior studies comparing survival to hospital-discharge rates between sexes in OHCA patients have not detected a difference between men or women after controlling for confounders.^2^,^12^,^13^ Ours is the first study to evaluate the effect of single dose vs. multi-dose epinephrine on subgroups by sex. In our study, rates of ROSC decreased post-implementation in both male and female subgroups without a significant difference between sexes. Despite this decrease in ROSC rates, SHD rates were similar between the single dose and multi-dose epinephrine groups in both sexes. Thus, our findings add to existing literature indicating that a difference in OHCA outcomes driven by sex is unlikely.

Our exploratory outcome of favorable neurologic status remained unchanged post-implementation in all subgroups. Although the PARAMEDIC-2 trial found similar rates of favorable neurologic outcomes between the epinephrine and placebo groups overall, severe neurologic disability was more common in the epinephrine group.^6^ It is possible that a dose-dependent relationship with epinephrine drives neurologic outcomes. Therefore, we theorized that single dose epinephrine patients might have improved neurologic outcomes compared to multi-dose epinephrine patients. However, we did not observe this in our study. Importantly, our study was not powered to examine for differences in favorable neurologic outcomes. Due to the modest sample size, we were unable to perform fully adjusted analyses for favorable neurologic outcomes in the race and sex subgroups. The ongoing Epinephrine Dose: Optimal vs. Standard Evaluation randomized controlled trial and other OHCA resuscitation studies may help us understand the relationship between epinephrine and meaningful neurologic recovery.

## LIMITATIONS

This study has limitations. First, the five participating EMS systems are all within North Carolina, limiting geographic generalizability. Next, use of the pre-post implementation study design exposed the study to unknown confounders that would not occur in randomized controlled trials. The CARES database does not contain the number of epinephrine doses administered or the time to administration. Nor is this information available from the local EMS agencies. Because our dataset was completely deidentified, we were unable to link encounters to the CARES registry or perform additional prehospital EHR review. Therefore, we were unable to restrict the cohort to only those who received epinephrine, account for protocol violations, or determine which patients received the correct dose of epinephrine. Therefore, we were unable to conduct a per protocol analysis.

Additionally, CARES outcomes are not adjudicated, thus risking misclassification bias. It is also possible that some patients identified as multiracial or transgender, thus further risking misclassification bias. Although misclassification bias regarding race is often a concern, the race data in CARES was recently validated in a large cohort of nearly 25,000 patients. Investigators found a high degree of concordance between race data in the CARES database and Medicare data.^22^ Therefore, using CARES to determine a patient’s race is reasonable. Due to the modest sample size, we analyzed race as a dichotomous variable. This may have masked variation between race subgroups. However, making race a two- or three-level variable is a common statistical approach in studies with modest sample size.^24^–^28^ Lastly, tests for interactions and within subgroups may be underpowered given the study’s sample size. This may have limited our ability to detect meaningful differences between groups, particularly among non-White patients given the smaller sample size.

## CONCLUSION

Implementation of a single dose epinephrine protocol was associated with similar survival to hospital-discharge rates among men, women, and non-White patients compared to the multi-dose epinephrine. However, survival to hospital-discharge rates did improve among White patients. In the subgroup of patients thought to have arrested from a primary cardiac etiology, we noted that survival to hospital-discharge rates improved with the single dose epinephrine protocol among non-White patients but were similar among men, women, and White patients. These differences are likely driven by unmeasured confounders or inadequate power. We do not believe there is a physiologic basis for the difference in outcomes between White and non-White patients with single dose epinephrine implementation. Future single dose epinephrine studies should continue to evaluate and monitor for potential OHCA care disparities and leverage larger cohorts so that more granular race findings can be reported.

## Supplementary Information





## Figures and Tables

**Table 1:** Cohort characteristics pre- vs post-implementation categorized by White vs non-White and male vs. female patients.

	Pre-Implementation MDEP (N = 899), n (%)		Post-Implementation SDEP (N = 791), n (%)	

Race	White n = 673 (%)	Non-White n = 226 (%)	White n = 589 (%)	Non-White n = 202 (%)
Age - median (IQR) (years)	65 (51–76)	64 (54–75)	66 (54–78)	63 (51–74)
Female	256 (38.0)	108 (47.7)	208 (35.3)	85 (42.1)
Race				
White	673 (100.0)	0 (0)	589 (100.0)	0 (0)
Black	0 (0)	213 (94.3)	0 (0)	179 (88.6)
Other	0 (0)	13 (5.8)	0 (0)	23 (11.4)
Hispanic/Latino	0 (0)	5 (2.2)	0 (0)	14 (6.9)
Forsyth County	289 (42.9)	172 (76.1)	228 (38.7)	143 (70.8)
Iredell County	107 (15.9)	26 (11.5)	103 (17.5)	32 (15.8)
Randolph County	162 (24.1)	17 (7.5)	158 (26.8)	20 (9.9)
Stanly County	24 (3.6)	4 (1.8)	29 (4.9)	5 (2.5)
Surry County	91 (13.5)	7 (3.1)	71 (12.1)	2 (1.0)
Cardiac	520 (77.3)	191 (84.5)	482 (81.8)	168 (83.2)
Non-cardiac	153 (22.7)	35 (15.5)	107 (18.2)	34 (16.8)
Respiratory	72 (10.7)	31 (13.7)	67 (11.4)	30 (14.9)
Overdose	71 (10.5)	3 (1.3)	35 (5.9)	2 (0.1)
Drowning	2 (0.3)	0 (0.0)	0 (0.0)	0 (0.0)
Electrocution	1 (0.1)	0 (0.0)	1 (0.2)	1 (0.5)
Other	7 (1.0)	1 (0.004)	4 (0.7)	1 (0.5)
Shockable	104 (15.5)	40 (17.7)	138 (23.4)	42 (20.8)
Ventricular fibrillation	71 (10.5)	25 (11.1)	90 (15.3)	23 (11.4)
Ventricular tachycardia	8 (1.2)	3 (1.3)	1 (0.2)	5 (2.5)
Other shockable rhythm	25 (3.7)	12 (5.3)	47 (8.0)	14 (6.9)
Non-shockable	569 (84.5)	186 (82.3)	451(76.6)	160 (79.2)
Asystole	380 (56.5)	102 (45.1)	274 (46.5)	91 (45.0)
Pulseless electrical activity	118 (17.5)	64 (28.3)	118 (20.0)	53 (26.2)
Other non-shockable rhythm	71 (2.5)	20 (8.8)	59 (10.0)	16 (7.9)
Witnessed cardiac arrest	390 (58.0)	127 (56.2)	360 (61.1)	107 (53.0)
Bystander CPR	278 (41.3)	88 (38.9)	217 (36.8)	75 (37.1)
AED available	66 (9.8)	20 (8.8)	85 (14.4)	19 (9.4)
Response interval (median, IQR) (minutes)	8.4 (6.1–11.4)	7.5 (5.5–9.6)	8.2 (5.7–10.7)	6.9 (5.1–9.2)

	Pre-Implementation MDEP (N = 899), n (%)		Post-Implementation SDEP (N = 791), n (%)	

Sex	Male n=535 (%)	Female n=364 (%)	Male n=498 (%)	Female n=293 (%)

Age (median, IQR) (years)	65 (52–75)	64.5 (50.5–77)	65 (52–77)	66 (54–78)
Female	0 (0)	364 (100)	0 (0)	293 (100)
White	417 (77.9)	256 (70.3)	381 (76.5)	208 (71.0)
Black	108 (20.2)	105 (28.9)	102 (20.5)	77 (26.3)
Other	10 (1.9)	3 (0.8)	15 (3.0)	8 (2.7)
Hispanic/Latino	4 (0.8)	1 (0.3)	11 (2.2)	3 (1.0)
Forsyth County	270 (50.4)	191 (52.5)	226 (45.4)	145 (49.5)
Iredell County	85 (15.9)	48 (13.2)	91 (18.3)	44 (15.0)
Randolph County	112 (20.9)	67 (18.4)	114 (22.9)	64 (21.8)
Stanly County	15 (2.8)	13 (3.6)	19 (3.8)	15 (5.1)
Surry County	52 (9.9)	45 (12.4)	48 (9.6)	25 (8.5)
Cardiac	428 (80.0)	283 (77.8)	411 (82.5)	239 (81.6)
Non-cardiac	107 (20.0)	81 (22.2)	87 (17.5)	54 (18.4)
Respiratory	56 (10.5)	47 (12.9)	51 (10.3)	46 (15.7)
Overdose	47 (8.8)	27 (7.4)	30 (6.0)	7 (2.4)
Drowning	1 (0.2)	1 (0.3)	0 (0)	0 (0)
Electrocution	1 (0.2)	0 (0)	2 (0.4)	0 (0)
Other	2 (0.4)	6 (1.7)	4 (0.8)	1 (0.3)
Shockable	103 (19.3)	41 (11.3)	124 (24.9)	56 (19.1)
Ventricular fibrillation	71 (13.3)	25 (6.9)	75 (7.3)	38 (13.0)
Ventricular tachycardia	4 (0.8)	7 (1.9)	3 (0.3)	3 (1.0)
Other shockable rhythm	28 (5.2)	9 (2.5)	46 (9.3)	15 (5.1)
Non-shockable	432 (80.7)	323 (88.7)	374 (75.1)	237 (80.9)
Asystole	276 (51.6)	206 (56.6)	234 (47.0)	131 (44.7)
Pulseless electrical activity	106 (19.8)	76 (20.9)	90 (18.1)	81 (27.7)
Other non-shockable rhythm	50 (9.4)	41 (11.3)	50 (10.0)	25 (8.5)
Witnessed cardiac arrest	315 (58.9)	202 (55.5)	293 (58.8)	174 (59.4)
Bystander CPR	225 (42.1)	141 (38.7)	188 (37.8)	104 (35.5)
AED available	66 (12.3)	20 (5.5)	77 (15.5)	27 (9.2)
Response interval (median, IQR) (minutes)	8.3 (5.9–11.2)	7.8 (5.8–10.3)	7.7 (5.4–10.4)	8.0 (5.7–10.6)

All rows show n (%) unless otherwise indicated.

*MDEP*, multidose epinephrine protocol; *SDEP*, single dose epinephrine protocol; *IQR*, interquartile range; *CPR*, cardiopulmonary resuscitation; *AED*, automatic external defibrillator.

*MDEP*, multidose epinephrine protocol; *SDEP*, single-dose epinephrine protocol; *IQR*, interquartile range; *CPR*, cardiopulmonary resuscitation; *AED*, automated external defibrillator.

**Table 2:** Race subgroups.

	White				Non-White				Interaction (Implementation cohort x race)
	
	Pre-implementation MDEP (N = 673) n (%)	Post-implementation SDEP (N = 589) n (%)	Odds Ratio (95% CI)		Pre-implementation MDEP (N = 226) n (%)	Post-implementation SDEP (N = 202) n (%)	Odds Ratio (95% CI)		
			Unadjusted	Adjusted^1^			Unadjusted	Adjusted^1^	
ROSC	298 (44.3)	193 (32.8)	**0.62 (0.43–0.89)**	**0.49 (0.33–0.75)**	82 (36.3)	64 (31.7)	0.83 (0.44–1.56)	0.82 (0.47–1.41)	0.13
SHD	89 (13.2)	89 (15.1)	**1.18 (1.06–1.31)**	**1.17 (1.05–1.30)**	33 (14.6)	33 (16.3)	1.17 (0.68–2.02)	1.08 (0.78–1.51)	0.84
Favorable neurologic outcome	77 (11.4)	63 (10.7)	0.91 (0.69–1.21)	1.07 (0.89–1.28)^2^	22 (9.7)	22 (10.9)	1.11 (0.65–1.89)	1.11 (0.66–1.88)^2^	0.39

1 djusted for age, sex (male vs female), witnessed arrest (yes/no), location of the arrest (home, medical facility, other), automatic external defibrillator availability (yes/no), emergency medical services response interval, the presence of a shockable rhythm (yes/no), and bystander cardiopulmonary resuscitation (yes/no).

2 djusted only for age and sex given the number of events.

*MDEP*, multidose epinephrine protocol; *SDEP*, single-dose epinephrine protocol; *ROSC*, return of spontaneous circulation; *SHD*, survival to hospital discharge.

**Table 3:** Sex subgroups.

	Male				Female				Interaction (Implementation cohort x sex)
	
	Pre-implementation MDEP (n = 535), n (%)	Post-implementation SDEP (n = 498), n (%)	Odds Ratio (95%CI)		Pre-implementation MDEP (n = 364), n (%)	Post-implementation SDEP (n = 293), n (%)	Odds Ratio (95% CI)		
			Unadjusted	Adjusted^1^			Unadjusted	Adjusted^1^	
ROSC	216 (40.4)	148 (29.7)	0.63 (0.40–0.997)	**0.54 (0.35–0.83)**	164 (45.1)	109 (37.2)	**0.72 (0.56–0.94)**	**0.61 (0.45–0.83)**	0.47
SHD	72 (13.5)	73 (14.7)	1.11 (0.97–1.28)	1.03 (0.93–1.14)	50 (13.7)	49 (16.7)	1.28 (0.91–1.80)	1.23 (0.97–1.56)	0.31
Favorable Neurologic Outcome	60 (11.2)	48 (9.6)	0.84 (0.61–1.16)	0.89 (0.65–1.21)^2^	39 (10.7)	37 (12.6)	1.21 (0.76–1.93)	1.36 (0.91–2.05)^2^	0.18

1 djusted for age, race (White vs non-White), witnessed arrest (yes/no), location of the arrest (home, medical facility, other), automatic external defibrillator availability, emergency medical services response interval, the presence of a shockable rhythm, receiving bystander cardiopulmonary resuscitation.

2 djusted only for age and race given the number of events.

*MDEP*, multi-dose epinephrine protocol; *SDEP*, single dose epinephrine protocol; *ROSC*, return of spontaneous circulation; *SHD*, survival to hospital discharge.

## References

[1] Sasson C, Rogers MAM, Dahl J (2010). Predictors of survival from out-of-hospital cardiac arrest: a systematic review and meta-analysis. Circ Cardiovasc Qual Outcomes.

[2] Virani SS, Alonso A, Aparicio HJ (2021). Heart disease and stroke statistics-2021 update: a report from the American Heart Association. Circulation.

[3] Lee SY, Song KJ, Shin SD (2018). A disparity in outcomes of out-of-hospital cardiac arrest by community socioeconomic status: a ten-year observational study. Resuscitation.

[4] Panchal AR, Bartos JA, Cabañas JG (2020). Part 3: Adult Basic and Advanced Life Support: 2020 American Heart Association Guidelines for Cardiopulmonary Resuscitation and Emergency Cardiovascular Care. Circulation.

[5] Jacobs IG, Finn JC, Jelinek GA (2011). Effect of adrenaline on survival in out-of-hospital cardiac arrest: a randomised double-blind placebo-controlled trial. Resuscitation.

[6] Perkins GD, Ji C, Deakin CD (2018). A randomized trial of epinephrine in out-of-hospital cardiac arrest. N Engl J Med.

[7] Ng KT, Teoh WY (2019). The effect of prehospital epinephrine in out-of-hospital cardiac arrest: a systematic review and meta-analysis. Prehosp Disaster Med.

[8] Overgaard CB, Dzavík V (2008). Inotropes and vasopressors: review of physiology and clinical use in cardiovascular disease. Circulation.

[9] Ristagno G, Tang W, Huang L (2009). Epinephrine reduces cerebral perfusion during cardiopulmonary resuscitation. Crit Care Med.

[10] Callaway CW (2013). Epinephrine for cardiac arrest. Curr Opin Cardiol.

[11] Ashburn NP, Beaver BP, Snavely AC (2023). One and done epinephrine in out-of-hospital cardiac arrest? Outcomes in a multiagency United States study. Prehosp Emerg Care.

[12] Ng YY, Wah W, Liu N (2016). Associations between gender and cardiac arrest outcomes in Pan-Asian out-of-hospital cardiac arrest patients. Resuscitation.

[13] Dicker B, Conaglen K, Howie G (2018). Gender and survival from out-of-hospital cardiac arrest: a New Zealand registry study. Emerg Med J.

[14] Zhao D, Post WS, Blasco-Colmenares E (2019). Racial differences in sudden cardiac death. Circulation.

[15] Mehta NK, Allam S, Mazimba S (2022). Racial, ethnic, and socioeconomic disparities in out-of-hospital cardiac arrest within the United States: Now is the time for change. Heart Rhythm O2.

[16] Monlezun DJ, Samura AT, Patel RS (2021). Racial and socioeconomic disparities in out-of-hospital cardiac arrest outcomes: artificial intelligence-augmented propensity score and geospatial cohort analysis of 3,952 patients. Cardiol Res Pract.

[17] Javed Z, Haisum Maqsood M, Yahya T (2022). Race, racism, and cardiovascular health: applying a social determinants of health framework to racial/ethnic disparities in cardiovascular disease. Circ Cardiovasc Qual Outcomes.

[18] Churchwell K, Elkind MSV, Benjamin RM (2020). Call to action: structural racism as a fundamental driver of health disparities: a presidential advisory from the American Heart Association. Circulation.

[19] von Elm E, Altman DG, Egger M (2007). The Strengthening the Reporting of Observational Studies in Epidemiology (STROBE) Statement: guidelines for reporting observational studies.

[20] George TS, Ashburn NP, Snavely AC (2025). Does single dose epinephrine improve outcomes for patients with out-of-hospital cardiac arrest and bystander CPR or a shockable rhythm?. Prehosp Emerg Care.

[21] Cardiac Arrest Registry to Enhance Survival (CARES) (2021). 2021 Data Dictionary.

[22] Chan PS, Merritt R, Chang A (2022). Race and ethnicity data in the cardiac arrest registry to enhance survival: Insights from Medicare self-reported data. Resuscitation.

[23] Worster A, Bledsoe RD, Cleve P (2005). Reassessing the methods of medical record review studies in emergency medicine research. Ann Emerg Med.

[24] Becker LB, Han BH, Meyer PM (1993). Racial differences in the incidence of cardiac arrest and subsequent survival. The CPR Chicago Project. N Engl J Med.

[25] Garcia RA, Spertus JA, Girotra S (2022). Racial and ethnic differences in bystander CPR for witnessed cardiac arrest. N Engl J Med.

[26] Veasey CJ, Snavely AC, Kearns ZL (2024). The high-sensitivity HEART pathway safely reduces hospitalizations regardless of sex or race in a multisite prospective US cohort. Clin Cardiol.

[27] Popp LM, Ashburn NP, Snavely AC (2023). Race differences in cardiac testing rates for patients with chest pain in a multisite cohort. Acad Emerg Med.

[28] Supples MW, Snavely AC, O’Neill JC (2024). Sex and race differences in the performance of the European Society of Cardiology 0/1-h algorithm with high-sensitivity troponin T. Clin Cardiol.

