# Supplementary material for: Does Single Dose Epinephrine Improve Outcomes for Patients with Out-of-Hospital Cardiac Arrest by Sex or Race?
Source: West J Emerg Med. 2025 Sep 25;26(5):1313–21. doi: 10.5811/westjem.41482 (PMC12591646; doi:10.5811/westjem.41482)
Supplement: Supplementary file 1 [file wjem-26-1313-s001.pdf]

Pre-Implementation Guideline-Based Multidose Epinephrine Protocol

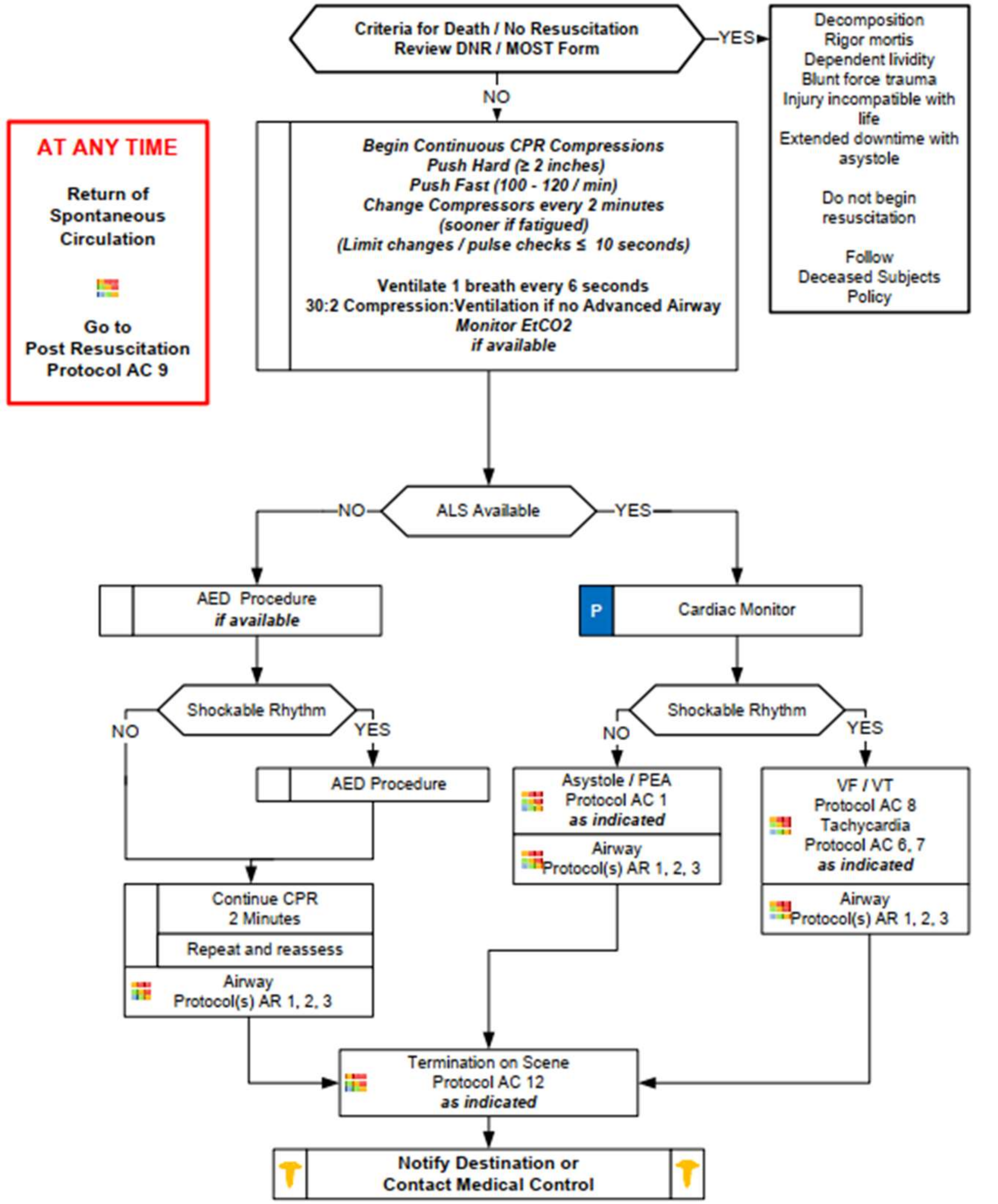

Follow Cardiac Arrest; Protocol AC3 and Team Focused CPR Protocol AC 11 and Termination of Resuscitation On Scene Protocol AC 12.

Primary focus is on high-quality, continuous and uninterrupted compressions at a rate of:

- 100 – 120 / minute, = 2 inches depth of compression, allow complete recoil of chest on upstroke.
- Do not interrupt compressions for more than 5 seconds optimal and 10 seconds maximum.
- Compressor counts aloud every 20th compression and next compressor readies themselves at the 180th compression.
- Paramedic should charge the defibrillator at the 180th compression.
- Compressor-on-deck ensures high-quality CPR visually and by monitor. Everyone on scene is responsible for ensuring high-quality, uninterrupted chest compressions.

Utilize respiratory counter device to ensure NO HYPERVENTILATION. Ventilations are to be delivered every 6 seconds.

When faced with either PEA or Asystole the most important aspect is finding a reversible cause.

Medication Sequence:
FIRST DOSE: Give Epinephrine 1mg (1:10,000)
SECOND DOSE: After 5 minutes of CPR - Give Epinephrine 1mg (1:10,000)
REPEAT DOSE: After 10 minutes of CPR give Epinephrine 1mg (1:10,000) as indicated below:

Every 5 minutes if EtCO2 < 30
Every 10 minutes if EtCO2 ≥ 30

When EtCO2 remains < 20, ensure high-quality compressions with proper depth, rate, and equal downstroke and upstroke.

Hyperkalemia: Unknown in field setting. End stage renal dialysis patient is at risk and Sodium bicarbonate and Calcium chloride should be given. ECG findings may not reflect common teaching such as peaked T waves. PEA with a bizarre or widened complex may indeed be hyperkalemia.

Post-Implementation Single Dose Epinephrine Protocol

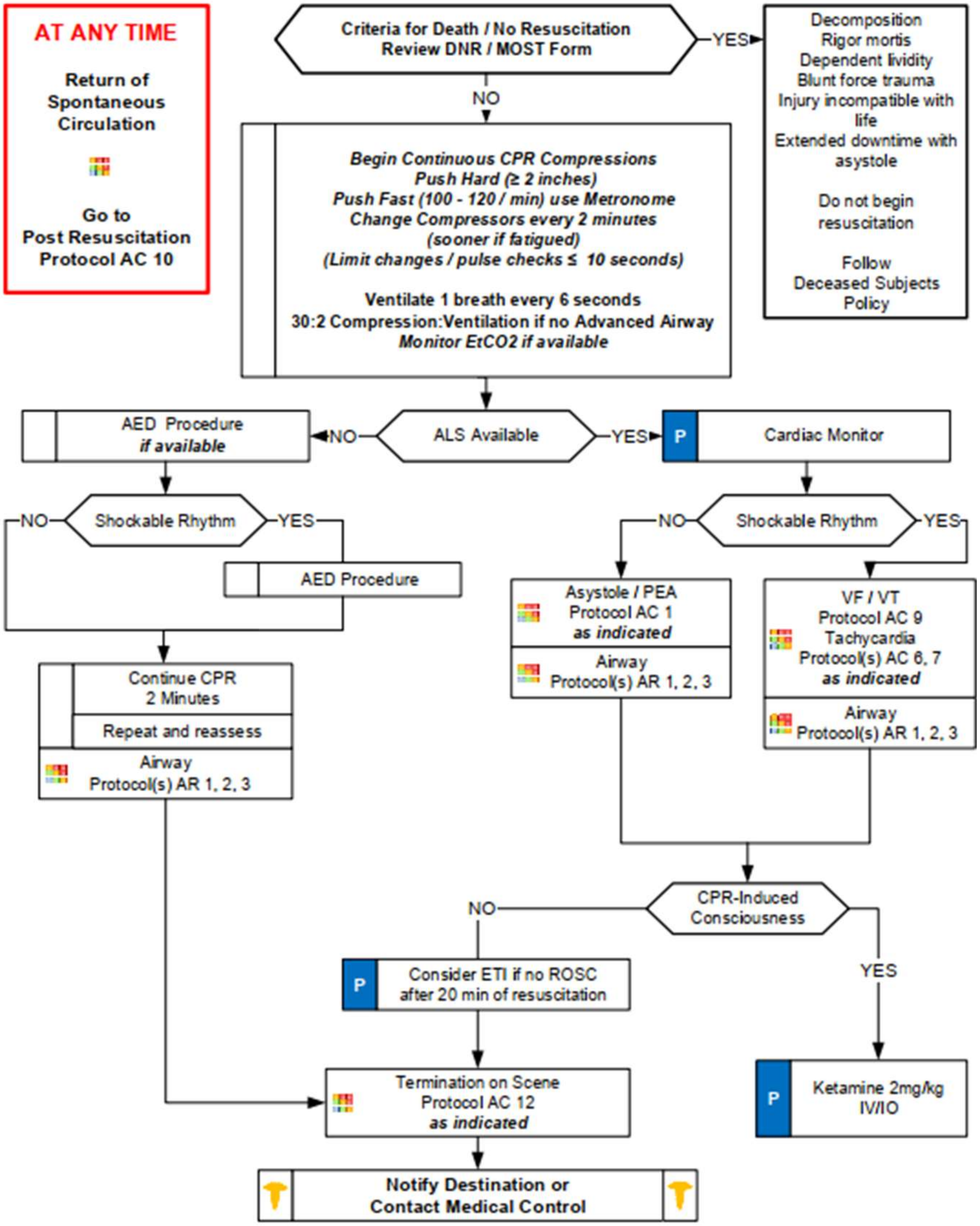

Follow Cardiac Arrest; Protocol AC3 and Team Focused CPR Protocol AC 11 and Termination of Resuscitation On Scene Protocol AC 12.

Primary focus is on high-quality, continuous and uninterrupted compressions at a rate of:

100-120 / minute, 2 inches depth of compression, allow complete recoil of chest on upstroke.
Do not interrupt compressions for more than 10 seconds maximum, 5 seconds if possible.

Compressor counts aloud q20th compression and next compressor moves in position at the 180th compression.
Ventilator provides ventilation breath every 20th compression via BVM, mouth-to-mask, BIAD, or ETT.
Paramedic should charge the defibrillator at the 180th compression.

When faced with either PEA or Asystole the most important aspect is finding a reversible cause.

Consider if this a cardiac event or a primary respiratory event, drug overdose, drowning, hanging, suffocation or trauma?

Medication Sequence:
SINGLE DOSE EPI: Give Epinephrine 1mg (1:10,000) IV/IO
Atropine not likely beneficial and no longer indicated with PEA or Asystole (can give at discretion of team leader to max of 3 mg.)
Hyperkalemia: Unknown in field setting. End stage renal dialysis patient is at risk and Sodium bicarbonate and Calcium chloride should be given. ECG findings may not reflect common findings such as peaked T waves. PEA with a bizarre or widened complex may indicate hyperkalemia.
