# Supplementary material for: Does Single Dose Epinephrine Improve Outcomes for Patients with Out-of-Hospital Cardiac Arrest by Sex or Race?
Source: West J Emerg Med. 2025 Sep 25;26(5):1313–21. doi: 10.5811/westjem.41482 (PMC12591646; doi:10.5811/westjem.41482)
Supplement: Supplementary file 2 [file wjem-26-1313-s002.docx]

**SUPPLEMENTAL TABLES AND FIGURES**

**Supplemental Table 1**. Description of each participating county and the protocol switch date.

| **County** | **Population** | **Rural/Urban** | **Single Dose Epinephrine Protocol Initiation Date** |
| --- | --- | --- | --- |
| Forsyth | 382,000 | Urban | 3/1/2018 |
| Iredell | 182,000 | Mixed | 11/1/2018 |
| Randolph | 144,000 | Mixed | 11/1/2017 |
| Stanly | 62,000 | Rural | 7/1/2018 |
| Surry | 72,000 | Rural | 3/1/2018 |

**Supplemental Table 2.** Race subgroups among patients thought to have died from a primary cardiac etiology.

|  | **White** | | | | **Non-White** | | | | **Interaction (Implementation cohort x race)** |
| --- | --- | --- | --- | --- | --- | --- | --- | --- | --- |
|  | Pre-implementation (n=520), n (%) | Post-implementation (n=482), n (%) | Odds Ratio (95%CI) | | Pre-implementation (n=191), n (%) | Post-implementation (n=168), n (%) | Odds Ratio (95%CI) | |  |
|  |  |  | Unadjusted | Adjusted^1^ |  |  | Unadjusted | Adjusted^1^ |  |
| **ROSC** | 208 (40.0) | 148 (30.7) | **0.66 (0.46-0.97)** | **0.49 (0.34-0.74)** | 64 (33.5) | 53 (31.6) | 0.93 (0.57-1.51) | 0.94 (0.69-1.30) | 0.15 |
| **SHD** | 49 (9.4) | 63 (13.1) | **1.42 (1.18-1.71)** | **1.28 (0.84-1.95)** | 21 (11.0) | 23 (13.7) | 1.25 (0.91-1.73) | **1.22 (1.01-1.48)** | 0.99 |
| **Favorable Neurologic Outcome** | 39 (7.5) | 43 (8.9) | 1.23 (1.02-1.49) | NA^2^ | 15 (7.9) | 16 (9.5) | 1.20 (0.78-1.85) | NA^2^ | 0.94 |

MDEP- multidose epinephrine protocol, SDEP – single dose epinephrine protocol, ROSC – return of spontaneous circulation, SHD – survival to hospital discharge

^1^ Adjusted for age, sex (male vs. female), witnessed arrest (yes/no), location of the arrest (home, medical facility, other), AED availability (yes/no), EMS response interval, the presence of a shockable rhythm (yes/no), and bystander CPR (yes/no).

^2^ Unable to adjust due to the small number of events

**Supplemental Table 3.** Sex subgroups among patients thought to have died from a primary cardiac etiology.

|  | **Male** | | | | **Female** | | | | **Interaction (Implementation cohort x sex)** |
| --- | --- | --- | --- | --- | --- | --- | --- | --- | --- |
|  | Pre-implementation MDEP (n=428), n (%) | Post-implementation SDEP  (n=411), n (%) | Odds Ratio (95%CI) | | Pre-implementation MDEP (n=283), n (%) | Post-implementation SDEP  (n=239), n (%) | Odds Ratio (95%CI) | |  |
|  |  |  | Unadjusted | Adjusted^1^ |  |  | Unadjusted | Adjusted^1^ |  |
| **ROSC** | 160 (37.4) | 120 (29.2) | **0.69 (0.51-0.94)** | **0.59 (0.44-0.79)** | 112 (39.6) | 81 (33.9) | 0.78 (0.52-1.17) | **0.63 (0.39-0.997)** | 0.44 |
| **SHD** | 44 (10.3) | 52 (12.7) | 1.24 (0.95-1.62) | 1.17 (0.73-1.87) | 26 (9.2) | 34 (14.2) | **1.60 (1.07-2.37)** | 1.35 (0.97-1.91) | 0.56 |
| **Favorable Neurologic Outcome** | 36 (8.4) | 35 (8.5) | 1.02 (0.88-1.17) | NA^2^ | 18 (6.4) | 24 (10.0) | 1.64 (0.87-3.10) | NA^2^ | 0.20 |

MDEP- multidose epinephrine protocol, SDEP – single dose epinephrine protocol, ROSC – return of spontaneous circulation, SHD – survival to hospital discharge

^1^ Adjusted for age, race (White vs. non-White), witnessed arrest (yes/no), location of the arrest (home, medical facility, other), AED availability (yes/no), EMS response interval, the presence of a shockable rhythm (yes/no), and bystander CPR (yes/no).

^2^ Unable to adjust due to the small number of events


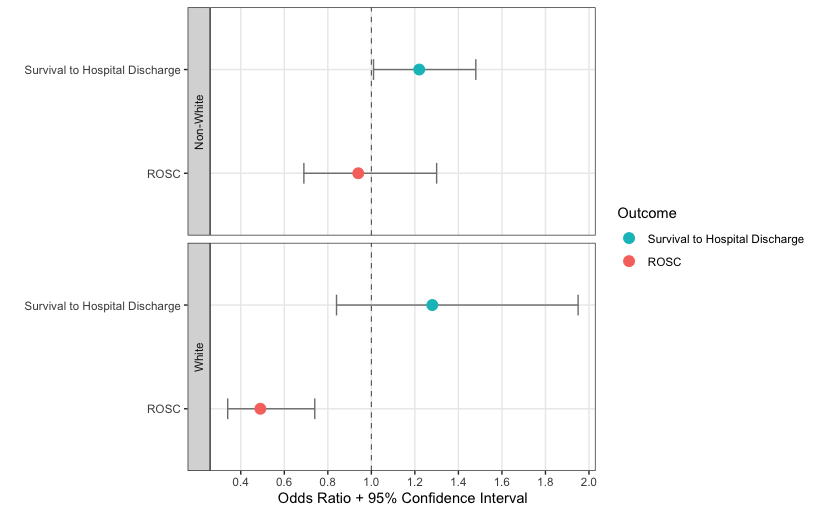


**Supplemental Figure 1**. SDEP vs. MDEP adjusted odds ratios for study outcomes among patients with presumed arrest from a cardiac etiology in White vs non-White patients. Models were adjusted for age, witnessed arrest, location of the arrest, AED availability, EMS response interval, the presence of a shockable rhythm, receiving bystander CPR, and sex or race.

MDEP- multidose epinephrine protocol, SDEP – single dose epinephrine protocol, ROSC – return of spontaneous circulation


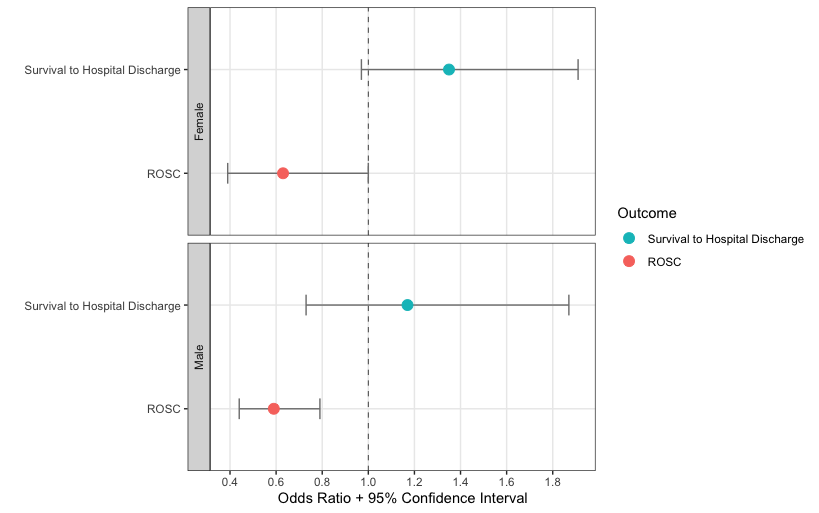


**Supplemental Figure 2.** SDEP vs. MDEP adjusted odds ratios for study outcomes among patients with presumed arrest from a cardiac etiology in male vs female patients. Models were adjusted for age, witnessed arrest, location of the arrest, AED availability, EMS response interval, the presence of a shockable rhythm, receiving bystander CPR, and sex or race.

MDEP- multidose epinephrine protocol, SDEP – single dose epinephrine protocol, ROSC – return of spontaneous circulation
